# The scope of mental health research during the COVID-19 pandemic and its aftermath

**DOI:** 10.1192/bjp.2020.125

**Published:** 2020-06-04

**Authors:** Matthew Hotopf, Ed Bullmore, Rory C. O'Connor, Emily A. Holmes

**Affiliations:** 1 Institute of Psychiatry Psychology and Neuroscience, King's College London; and National Institute of Health Research Biomedical Research Centre, South London and Maudsley NHS Foundation Trust, London, UK; 2 Department of Psychiatry, University of Cambridge; and Department of Research and Development, Cambridge and Peterborough NHS Foundation Trust, Cambridge, UK; 3 Suicidal Behaviour Research Laboratory, Institute of Health and Wellbeing, University of Glasgow, UK; 4 Department of Psychology, Uppsala University; and Department of Clinical Neuroscience, Karolinska Institutet, Stockholm, Sweden

**Keywords:** COVID-19, SARS-CoV-2, clinical neurology, psychosocial, psychosocial interventions

## Abstract

The effects of the COVID-19 pandemic on population mental health are unknown. We need to understand the scale of any such impact in different sections of the population, who is most affected and how best to mitigate, prevent and treat any excess morbidity. We propose a coordinated and interdisciplinary mental health science response.

It is self-evident that the COVID-19 pandemic has profound consequences for individuals and societies. Most research has understandably been focused on understanding the dynamics of the pandemic and the biology of the infection in order to develop diagnostics, vaccines and treatments. However, we know that, with time, the current spike of infections will pass. There will be headline mortality figures and infection rates, lessons learned about emergency preparedness, debates about the merits of competing strategies to control the infection, but as the rate of new infections continues to slow, schools will reopen and some semblance of normality will return. However, the impact of the pandemic on human health is likely to be felt for much longer than the first wave of severe illness and death.

## The long-term effects of the virus

### Neurological and psychiatric consequences

The long-term effects of SARS-CoV-2, the virus that causes COVID-19, on those who recover from the acute respiratory phase of COVID-19 are unknown. Just over one-third of patients with COVID-19 in Wuhan had neurological symptoms noted in their case notes, with anosmia and ageusia recognised as early symptoms unrelated to mucosal congestion.^1^ Other coronaviruses are neurotropic and capable of infecting neurons trans-synaptically. The immune response to infection could also have adverse effects on brain function. It is possible that infection or inflammation of homeostatic centres of respiration in the brain-stem may contribute to respiratory distress or failure. In the longer term, Parkinsonian symptoms were an important late complication of the 1918–1919 influenza pandemic, and neuropsychiatric complications may similarly arise following SARS-CoV-2 infection. As yet, virtually nothing is known of the neuropathology of the infection. However, even without direct effects on the brain, long-term mental health consequences might be anticipated. Mental disorders and cognitive impairments are common following treatment in intensive care. The systemic effects of infection, including cytokine storms as part of intense inflammatory or autoimmune response, combined with the mortal threat of the illness, constitute major biological and psychological stresses. It is plausible that there may be post-traumatic stress reactions, persistent fatigue, depression or physical symptoms of unclear aetiology as a chronic consequence of this acute combination of infection, health anxiety and heightened stress.

### Psychosocial impacts

However, for all of us – not just those infected or seriously ill – the psychosocial impacts of the pandemic are profound. The closure of schools, nurseries, pubs, shops, gyms and workplaces; the effect of self-isolation and loneliness, particularly for older people and those with multiple morbidities; the potential of self-isolation to exacerbate adverse home environments for children as well as adults, including domestic violence and abuse; the loss of employment, particularly for those with the most precarious working lives; the misinformation, confusion and, for some, anger around government policy; and the unprecedented restrictions on liberty resulting from public health measures to stem the pandemic affect every part of society, although those already disadvantaged will be most affected. We have already seen evidence of the pandemic having particularly adverse outcomes for people from Black and minority ethnic groups – these differential effects on mental and physical health need to be better understood. The longer-term impacts of a likely recession may ultimately have a more significant effect on health – particularly mental health – than the crisis itself. It is unknown whether or how these changes in our lives will affect mental health, and therefore research to monitor self-harm and suicide and the prevalence of mental and substance use disorders in the general population and populations at particular risk is vital.^2^

People with mental disorders may be particularly susceptible to these wider societal impacts. The anxieties associated with the pandemic may be more salient to people with pre-existing disorders – for example those with obsessive–compulsive disorders may be particularly affected by advice to hand-wash, or those with psychotic disorders may be more prone to subsume COVID-related threat preoccupations into delusional systems. There are significant challenges in delivering mental healthcare, particularly to those with the most severe difficulties, with the pandemic affecting already depleted staffing complements. Much routine mental healthcare has suddenly been curtailed or is now delivered remotely via video conferencing. What impact this has on the quality of patients’ care is unknown.

Meanwhile for the health and social care workforce, from the front-line staff in acute medicine and intensive care units to those helping frail elderly care-home residents, there are well-publicised challenges in terms of high job demands, lack of personal protective equipment, an atmosphere of heightened threat and potential ‘moral injury’ – the psychological impact of being forced to make hard choices that jar against the individual's ethical norms.^3^

## Questions for mental health research

A group convened by the mental health research charity MQ and the Academy of Medical Sciences has now developed a series of recommendations, published in *The Lancet Psychiatry*,^4^ to prioritise the research agenda for mental health science in the COVID-19 pandemic. The work of the group was informed by a rapid public consultation and collaboration with experts with lived experience and other research consumers, including senior clinicians.

Our single most important message is the need for high-quality mental health research as part of the wider research response to the pandemic. Nowhere is the case for integrating mental and physical health more pressing than in our response to the pandemic. There is a relatively short window of time in which to act. Existing infrastructure, data assets and expertise need to be mobilised, with funding put in place to respond. There are effectively three types of questions to answer, discussed in the paragraphs below, which apply to virtually every group affected by the pandemic. Who is most affected? Why and how are they affected? And what can be done to prevent, mitigate or treat problems faced by these groups?

### Who is most affected?

It is necessary first to understand the impact, if any, of the pandemic on various mental health outcomes across society. For the general population has there been an increase in suicide or self-harm, anxiety or depression? For people with pre-existing mental disorders, has the mortality gap widened as a result of their multiple disadvantages? For children and young people, has the prolonged period of school and university closure and uncertainty about exams affected their mental health? There is a need for epidemiologically robust methods – using either administrative data from health records (or similar systems) or by constructing new surveys. Although there have been many surveys delivered via social media, these are self-selecting and are likely to exaggerate health impacts. For the National Health Service (NHS), employers have a duty of care to understand the effects of work on their employees, much as the military does during and after deployment.

### Why and how are they affected?

We then need to understand mechanisms to explain why some individuals are more affected than others and how. That is, to be able to inform mental health interventions, the types of mechanism we are most interested in are those that are both causal and modifiable. These mechanisms will range from molecular and physiological to psychological and societal.^5^ In understanding long-term outcomes for people with severe COVID-19 illness, it will be necessary to resolve whether any effect on mental health arises from the possible neurotropic action of the virus, a more general impact of the ‘cytokine storm’ that accompanies severe systemic infection, or the alarming experience of being mortally ill, as related to post-traumatic stress reactions. We need to better understand the psychological mechanisms that account for changes such as anxiety, depression, self-harm and suicide more generally in the population. This understanding will inform the development of new mechanistically based psychological treatments that can be delivered under pandemic conditions.

### What can be done to intervene?

Next, there is a need to know what – if anything – should be done to intervene, bearing in mind that the current surge of cases of COVID-19 may be only the first in a series of spikes in incidence, and research conducted now may usefully inform responses to future waves of infection. Research can identify not only benefits of treatments, but also the harms of well-meaning interventions, for example debriefing following traumatic incidents. It is important that the pandemic response does not exacerbate existing social and health inequalities. The need to provide interventions at scale and remotely means that various modalities of digital intervention will inevitably dominate. Digital tools can operate on a spectrum from providing information to being used as a vehicle for delivering psychotherapies. We urge caution though, because only a few of the thousands of apps already available have a robust evidence base. It is vital that such tools are robustly evaluated before implementation.^5^ Randomisation and evaluation of competing digital systems could be a condition of any roll out in the NHS. Individual-based interventions are not the only approach and in many settings the response may be best conceptualised as a social one. Community-level interventions and volunteering that focus on altruism are an increasingly prominent response to the pandemic, which may have direct mental health benefits. For front-line healthcare workers structural responses relating to rotas and breaks, getting sufficient sleep, team working and practical support from employers may be more appropriate than individual-level interventions. Effective population health messaging – getting people to follow expert advice to reduce risk of COVID-19 infection – is critical not just for minimising anxiety that may otherwise contribute to mental health complications but more generally for boosting adherence to social distancing and other measures intended to slow transmission of the virus. We need to know this urgently to help in face of future waves of the pandemic.

## A coordinated research response

If research is to address the diverse challenges to mental health of the COVID-19 pandemic, several conditions need to be met. First, no single discipline will be able to address the problem alone. We call for mental health science, which we see as a multidisciplinary endeavour, combining psychiatry and psychology, neuroscience and neurology, social sciences, epidemiology and public health, and lived experience, to work together in responding to this crisis. Second, it is critical that there are open, two-way channels between consumers (patients, public and policy makers) and producers of mental health research, so that scientists are oriented to the questions that matter most to the public, and policy-making is properly informed by the best evidence available. Third, cooperation within and between sectors – research funders, universities, the NHS, industry and charities – is critical to the rapid and coherent deployment of existing infrastructure for mental health research. Fourth, definitive and impactful research will require national and international coordination, to ensure that protocols and computer code are shared, measures are harmonised, and work is done at scale, with the principles of open science at heart. Although we must act urgently, we must also be strategic and joined-up in how we address this challenge from the outset. Our success in understanding and mitigating the biological, psychological and social impacts of COVID-19 on mental health will require new investment from research funders and coordinated action from the entire community of mental health scientists.

## Supplementary material

For supplementary material accompanying this paper visit http://dx.doi.org/10.1192/bjp.2020.125. click here to view supplementary material
